# Diagnosis of focal liver lesions from ultrasound images using a pretrained residual neural network

**DOI:** 10.1002/acm2.14210

**Published:** 2023-11-22

**Authors:** Sutthirak Tangruangkiat, Napatsorn Chaiwongkot, Chayanon Pamarapa, Thanatcha Rawangwong, Araya Khunnarong, Chanyanuch Chainarong, Preeyanun Sathapanawanthana, Pantajaree Hiranrat, Ruedeerat Keerativittayayut, Witaya Sungkarat, Monchai Phonlakrai

**Affiliations:** ^1^ School of Radiological Technology, Faculty of Health Science Technology Chulabhorn Royal Academy Bangkok Thailand; ^2^ Sonographer School, Faculty of Health Science Technology Chulabhorn Royal Academy Bangkok Thailand

**Keywords:** convolutional neural network, focal liver lesions, hepatocellular carcinoma, ResNet50, ultrasound

## Abstract

**Objective:**

This study aims to develop a ResNet50‐based deep learning model for focal liver lesion (FLL) classification in ultrasound images, comparing its performance with other models and prior research.

**Methodology:**

We retrospectively collected 581 ultrasound images from the Chulabhorn Hospital's HCC surveillance and screening project (2010–2018). The dataset comprised five classes: non‐FLL, hepatic cyst (Cyst), hemangioma (HMG), focal fatty sparing (FFS), and hepatocellular carcinoma (HCC). We conducted 5‐fold cross‐validation after random dataset partitioning, enhancing training data with data augmentation. Our models used modified pre‐trained ResNet50, GGN, ResNet18, and VGG16 architectures. Model performance, assessed via confusion matrices for sensitivity, specificity, and accuracy, was compared across models and with prior studies.

**Results:**

ResNet50 outperformed other models, achieving a 5‐fold cross‐validation accuracy of 87 ± 2.2%. While VGG16 showed similar performance, it exhibited higher uncertainty. In the testing phase, the pretrained ResNet50 excelled in classifying non‐FLL, cysts, and FFS. To compare with other research, ResNet50 surpassed the prior methods like two‐layered feed‐forward neural networks (FFNN) and CNN+ReLU in FLL diagnosis.

**Conclusion:**

ResNet50 exhibited good performance in FLL diagnosis, especially for HCC classification, suggesting its potential for developing computer‐aided FLL diagnosis. However, further refinement is required for HCC and HMG classification in future studies.

## 1 INTRODUCTION

Hepatic cancer is a significant global health issue with rising incidence and mortality rates. It's most prevalent in East Asia and sub‐Saharan Africa due to limited access to medical resources.^1^ Prompt and precise diagnosis is crucial for better patient outcomes.^1^, ^2^ Imaging methods like ultrasound (US), computed tomography (CT), and magnetic resonance imaging (MRI) help detect and characterize liver cancers.^3^ US is often used initially because it is widely available and easy to use, especially for high‐risk populations like cirrhosis or chronic viral hepatitis patients.^4^, ^5^, ^6^ However, US has limitations compared to CT and MRI, such as operator dependence and reduced sensitivity for small lesions.^7^ Thus, choosing the right imaging method depends on clinical scenarios, with CT and MRI preferred for comprehensive evaluation.^2^, ^8^ Exciting advancements in US, like elastography and fusion imaging, may improve its diagnostic capabilities in the future.^9^

The emergence of artificial intelligence (AI) in recent years has opened doors to advanced computational tools that can assist radiologists in interpreting medical images and improving diagnostic accuracy.^10^ AI algorithms, particularly machine learning and deep learning models, are able to analyze large volumes of imaging data and extract meaningful patterns, achieving highly accurate and efficient identification and classification of liver lesions.^11^ The role of AI in medical imaging for diagnosing liver cancers holds immense potential for improving diagnostic accuracy and efficiency, leading to better patient outcomes.^11^ Deep learning techniques, such as convolutional neural networks (CNNs), have shown great promise in medical image analysis.^12^, ^13^, ^14^ ResNet50, a widely used CNN architecture, has demonstrated its effectiveness in diagnosing liver cancers from medical images.^15^, ^16^, ^17^ The incorporation of residual connections into RestNet50 allows this network to learn complex features from images, leading to improved classification accuracy.

Tiyarattanachai et al.^17^ developed a predictive model for detecting and classifying FLLs using RetinaNet. The study employed a diverse dataset of hepatic ultrasonography images, which demonstrated promising results indicative of high model performance. However, opportunities for improvement persist due to certain challenges. Notably, the presence of blood vessels within the liver, heterogeneous background liver parenchyma, renal cysts, inferior vena cava, and splenic lesions within the bounding box constraints have an adverse impact on the model's performance in FLL detection. This limitation was reflected in a lower detection rate in the external validation dataset (75.0%, 95%CI: 71.7−78.3). Schmauch et al.^18^ employed the ResNet50 model for the classification of focal liver lesions (FLLs) in ultrasound images. They designed a two‐stage approach for simultaneous FLL detection and classification, with model performance assessed using the Area Under the Curve (AUC) metric. Although the study demonstrated a notably high AUC value for FLL detection and classification, it is important to note that their dataset lacks ground truth information for lesion confirmation, and it contains a limited number of hepatocellular carcinoma (HCC) images (only 6). Mostafiz et al.^19^ introduced an innovative method for detecting FLLs in ultrasound images. Their approach integrates deep feature fusion and super‐resolution techniques, exhibiting promising advancements in lesion detection accuracy. However, it is imperative to recognize certain limitations that can provide guidance for future research initiatives. Notably, this approach is tailored for a binary classification model distinguishing normal liver tissue from FLLs.

Notwithstanding the above advancements provided by AI, this technology poses several challenges when applied to US image analysis that stem from the unique characteristics of US imaging. Only a few studies have examined the effectiveness of AI, and specifically RestNet50, for diagnosing liver cancer from ultrasound images. Previous studies have adopted a wide range of deep learning models and approaches, yielding inconsistent findings of AI models’ performance in diagnosis performance.^20^, ^21^, ^22^, ^23^, ^24^

This study proposes the integration of ResNet50, a CNN‐based model, to improve the diagnosis of focal liver lesions from ultrasound images. Our objective is to develop a deep learning model based on the ResNet50 architecture and assess its efficacy in classifying five liver tissue classes: non‐focal liver lesion (non‐FLL), simple hepatic cyst (Cyst), focal fatty sparing (FFS), hemangioma (HMG), and hepatocellular carcinoma (HCC). Additionally, we conduct a comparative analysis with different models and previous research findings to gain further insights into the diagnostic potential of ResNet50 for the identification of focal liver lesions.

## 2 MATERIALS AND METHODS

### 2.1 Study design

This retrospective study uses ultrasound images from patients that were eligible for the Hepatocellular Carcinoma (HCC) surveillance and screening initiative project at Chulabhorn Hospital between 2010 and 2018. All images were obtained in DICOM format from the Picture Archiving and Communication System (PACS). The objective of this study was to develop a model for classifying lesions from these images into HCC, HMG, FFS, Cyst, and non‐FLL. The findings of this study will serve as a foundation for the development of a computer‐aided diagnosis (CADx) system in the future.

### 2.2 Dataset

We retrospectively collected 581 B‐mode ultrasound images obtained with the upper abdominal ultrasonography protocol from 581 individual livers. Our inclusion criteria were: (1) participation in the Hepatocellular Carcinoma (HCC) surveillance and screening initiative project; (2) availability of CT and/or MRI confirmation of hepatic lesions. The dataset contained 150 images of non‐FLL, 150 images of Cyst, 77 images of FFS, 150 images of HMG, and 54 images of HCC. We anonymized the collected dataset. Our protocol was approved by the Human Research Ethics Committee of Chulabhorn Research Institute.

### 2.3 Ground truth

We relied on radiological reports from CT or MRI studies to validate the diagnosis of FLL. It is important to emphasize that CT and MRI are widely recognized as standard medical imaging modalities for diagnosing HCC, in accordance with the guidelines for HCC diagnosis in the diagnostic radiology workflow.^25^

### 2.4 Image preprocessing

**FIGURE 1:**
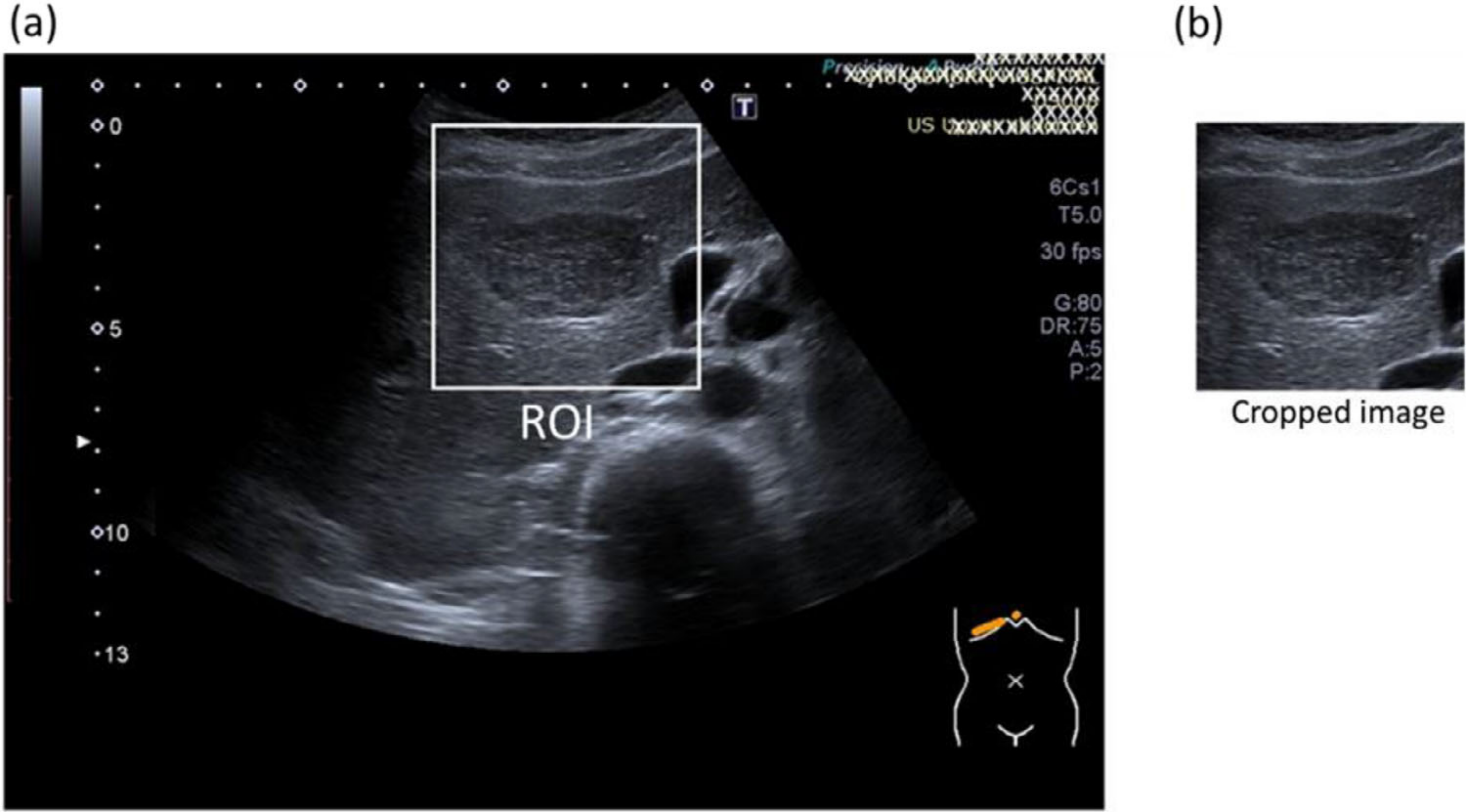
Illustrates ROI placement on a hemangioma in an ultrasound image (a) and cropped image (b).

An experienced sonographer manually cropped all liver lesions, with the size of the region‐of‐interest (ROI) adjusted to lesion size (see Figure 1). Non‐FLL regions were cropped using ROIs sized between 300 × 300 and 400 × 400 pixels. The resulting images were then resized to a standardized size of 224 × 224 pixels to meet the requirements of the input layer within the ResNet50 model. The original ultrasound images obtained from PACS are in RGB format with three color channels (red, green, and blue). The annotations, representing the selected areas, were displayed using color. In order to standardize the annotation process, this study converted the RGB ultrasound images to grayscale. All images were then converted back to RGB format using custom code running in Matlab (MATLAB® 2021b).

### 2.5 Data splitting and image augmentation

The dataset was categorized into five classes: non‐FFL, cyst, FFS, Hemangioma, and HCC. To obtain a sufficiently diverse dataset with an adequate number of samples for each class. To address the bias resulting from imbalanced training data, we implemented a preprocessing technique for classes with less than 150 images. This technique involved rescaling or translating the images to artificially increase their count to 150 for each class (750 images in total).

### 2.6 Five‐fold cross validation

In this study, we employed a five‐fold cross‐validation to assess the accuracy and generalizability of four distinct models: the graph‐generative neural network (GGN), ResNet18, ResNet50, and Visual Geometry Group (VGG16) models. This involved randomly and equally partitioning the dataset into five subsets and training/testing the models on different combinations of these subsets. In the training phase of the five‐fold cross‐validation, we enhanced the number of images by applying various image augmentation techniques to four subsets within each fold. These techniques included translating images along both the x‐axis and y‐axis, as well as horizontally flipping them. As a result, we significantly increased the number of images per subset from 150 to 600. This process yielded a total of 2400 images for training across the five‐fold. This augmentation aimed to enrich the training dataset and ensure a more balanced representation of the various classes during model training. Subsequently, we compared the performance of the ResNet50 model with that of the GGN, ResNet18, and VGG16 models. During the testing phase, we employed a set of 150 images from the remaining subset in each fold. This set comprised 30 images per class and was used to assess the performance of all the models.

**FIGURE 2:**
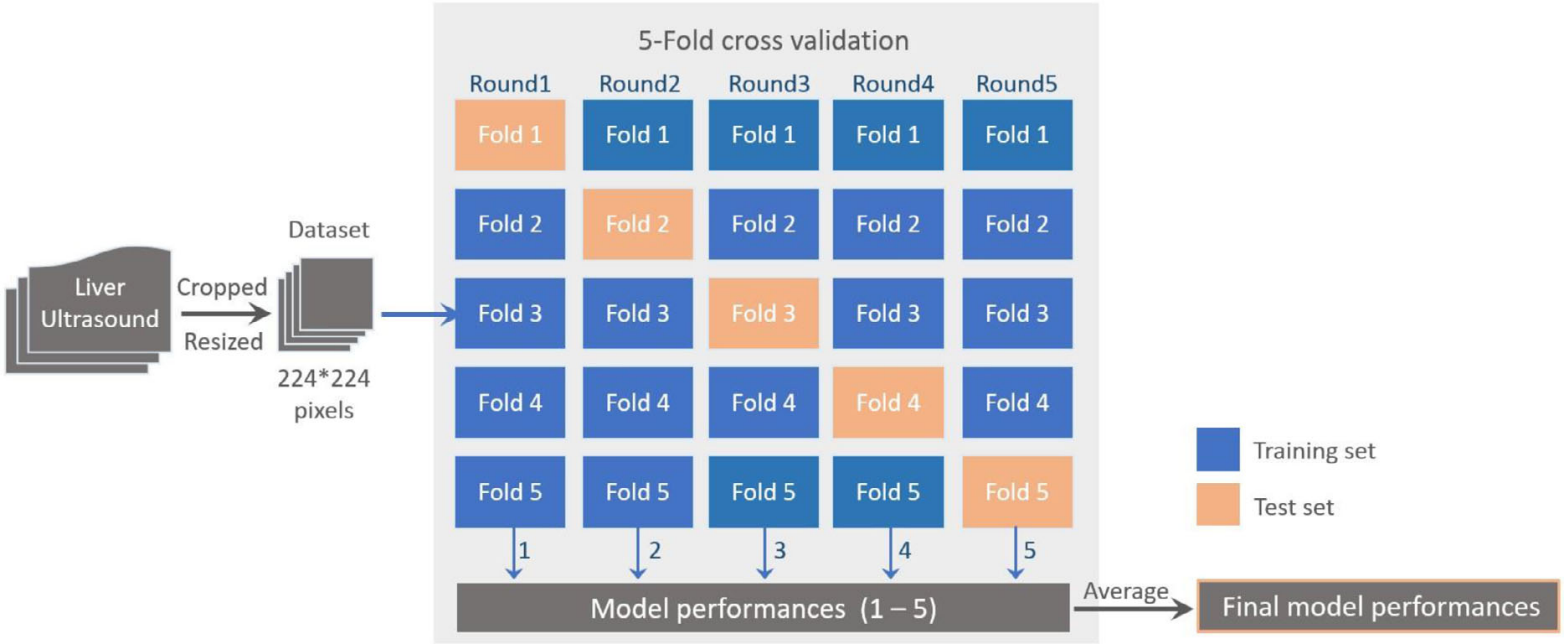
Depicts the workflow that accompanies our research methodology. This process encompasses several key stages. Initially, the dataset was randomly divided into five folds for the purpose of cross‐validation. Subsequently, the overall performance of the models was assessed through five rounds of separate training and testing.

Figure 2 illustrates the workflow of our approach, encompassing image preprocessing, data splitting, five‐fold cross‐validation, and model performance assessment.

### 2.7 CNN‐ResNet50 architecture

This study adopted and modified a preexisting pretrained convolutional neural network (CNN) for image recognition known as Residual Network 50 (ResNet50) to develop a diagnostic model for FLLs. Our modified model is based on the original ResNet50 architecture introduced by He et al. in 2015,^26^ comprising 50 layers. Our code for model development is available from GitHub: https://github.com/PinkSutthirak/classifier‐from‐restnet. ResNet50 introduced the concept of residual connections to address the degradation problem in deep neural networks, allowing for the training of much deeper models. ResNet50 has been widely used for various computer vision tasks, including image classification,^27^, ^28^ object detection,^29^, ^30^ and image segmentation.^31^ Its depth and residual connections allow it to learn intricate features and capture fine‐grained details, making it a powerful architecture for visual recognition tasks.

**FIGURE 3:**
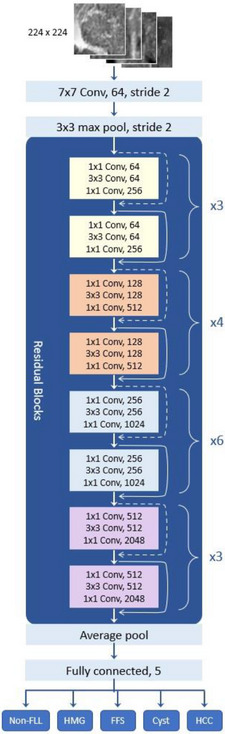
Illustrates the modified CNN‐ResNet50 architecture, which has been adapted from the original architecture to accommodate five classes of FLL.

Figure 3 illustrates the key layers of the ResNet50 architecture used in this study, including:

- Input layer: Accepts input images of size 224 × 224 pixels with RGB color channels. To align images with the prescribed specification of this layer, grayscale images were converted to RGB format.
- Convolutional layers: The initial layer applies 64 filters of size 7 × 7 with a stride of 2, extracting low‐level features from the input image.
- Max pooling layer: Reduces the spatial dimensions of the feature maps using a 3 × 3 pool size and a stride of 2.
- Residual blocks: ResNet50 incorporates a hierarchical structure with 16 residual blocks distributed across four stages. Each stage exhibits a varying number of blocks and filter sizes. Stage 1 encompasses three blocks: two convolutional layers with 64 filters, and one convolutional layer with 256 filters. Stage 2 comprises four blocks: two convolutional layers with 128 filters, and one convolutional layer with 512 filters. Stage 3 consists of six blocks: two convolutional layers with 256 filters, and one convolutional layer with 1024 filters. Lastly, stage 4 involves three blocks: two convolutional layers with 512 filters, and one convolutional layer with 2048 filters.
- Global average pooling: Converts 2D feature maps into 1D vectors by averaging across each feature map channel, resulting in fixed‐length feature vectors.
- Fully connected layers: Two fully connected layers with five units each serve as classifier, mapping the learned features to specific class labels. The final softmax activation layer converts the output of the fully connected layers into probabilities, representing predicted class probabilities for the input image.
- Output layer: This layer serves as classification output layer, estimating the likelihood or probability that the input data belong to one of the possible classes: non‐FFL, cyst, FFS, Hemangioma, or HCC.

### 2.8 Evaluation metrics for model performance

To evaluate the performance of the proposed method, we used confusion matrices to obtain the following three metrics, including accuracy, sensitivity, and specificity^19^:

#### 2.8.1 Accuracy

This metric describes the number of correct predictions over all predictions. The percentage of *Accuracy* is calculated using the following formula:

(1) $$\%Accuracy\;=\frac{\left(\mathrm{TP}\;+\;\mathrm{TN}\right)}{\left(\mathrm{TP}\;+\;\mathrm{FP}\;+\;\mathrm{FN}\;+\;\mathrm{TN}\right)}\;x\;100$$

where TP is True Positives, TN is True Negatives, TP is True Positive, and FP is False Positives.

#### 2.8.2 Sensitivity or recall or true positive rate (TPR)

This metric refers to the proportion of actual positive instances correctly identified by the model. It is calculated as the number of true positives divided by the sum of true positives and false negatives. Mathematically, the percentage of sensitivity can be expressed as:

(2) $$\%\mathrm{Sensitivity}\;=\frac{\mathrm{TP}\;}{\left(\mathrm{TP}\;+\mathrm{FN}\right)}\;x\;100$$

#### 2.8.3 Specificity

It refers to the ability of the deep learning model to correctly identify negative samples or true negatives. Specificity is calculated as the ratio of true negatives to the sum of true negatives and false positives:

(3) $$\%\mathrm{Specificity}\;=\frac{\mathrm{TN}\;}{\left(\mathrm{TN}\;+\mathrm{FP}\right)}\;x\;100$$

## 3 RESULTS

### 3.1 Implementation details

**FIGURE 4:**
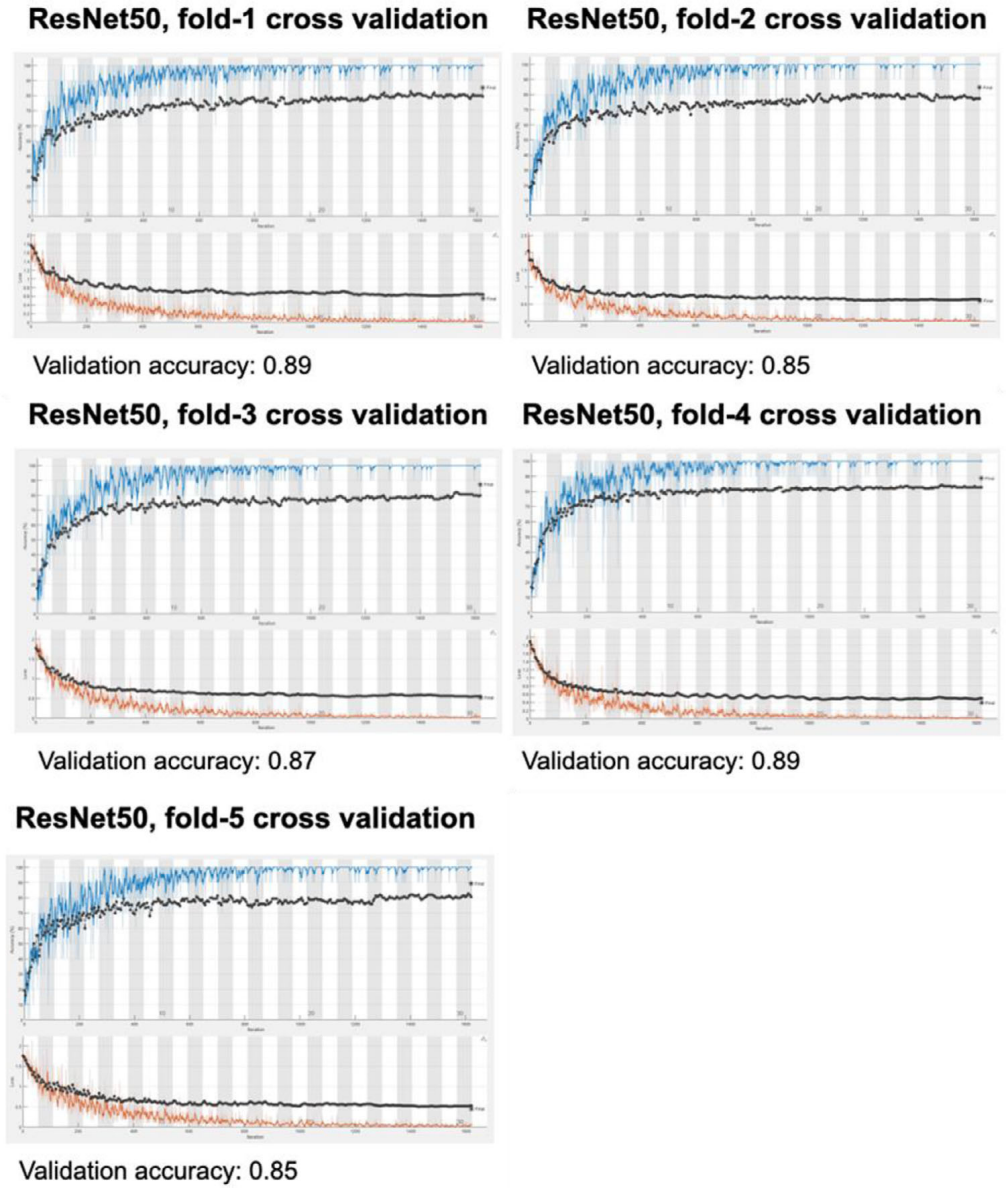
Depicts model accuracy (upper graph) and loss function (lower graph) after 30 epochs of 54 iterations each across 5‐fold cross validation. The ResNet50 model consistently achieved a validation accuracy ranging from 85.0% to 89.0%.

The study revealed that employing a 5‐fold strategy effectively eliminated overfitting in all models. Figure 4 demonstrates that ResNet50's training phase reached peak performance after 30 epochs. The output of the fully connected layer was transformed into five classes. Common hyperparameters were selected for all models, including the optimization algorithm (SGDM), MiniBatchSize (128), iterations per epoch (54), maximum epochs (30), learning rate (0.0001), and ValidationFrequency (50).

### 3.2 Model performances’ comparison

**FIGURE 5:**
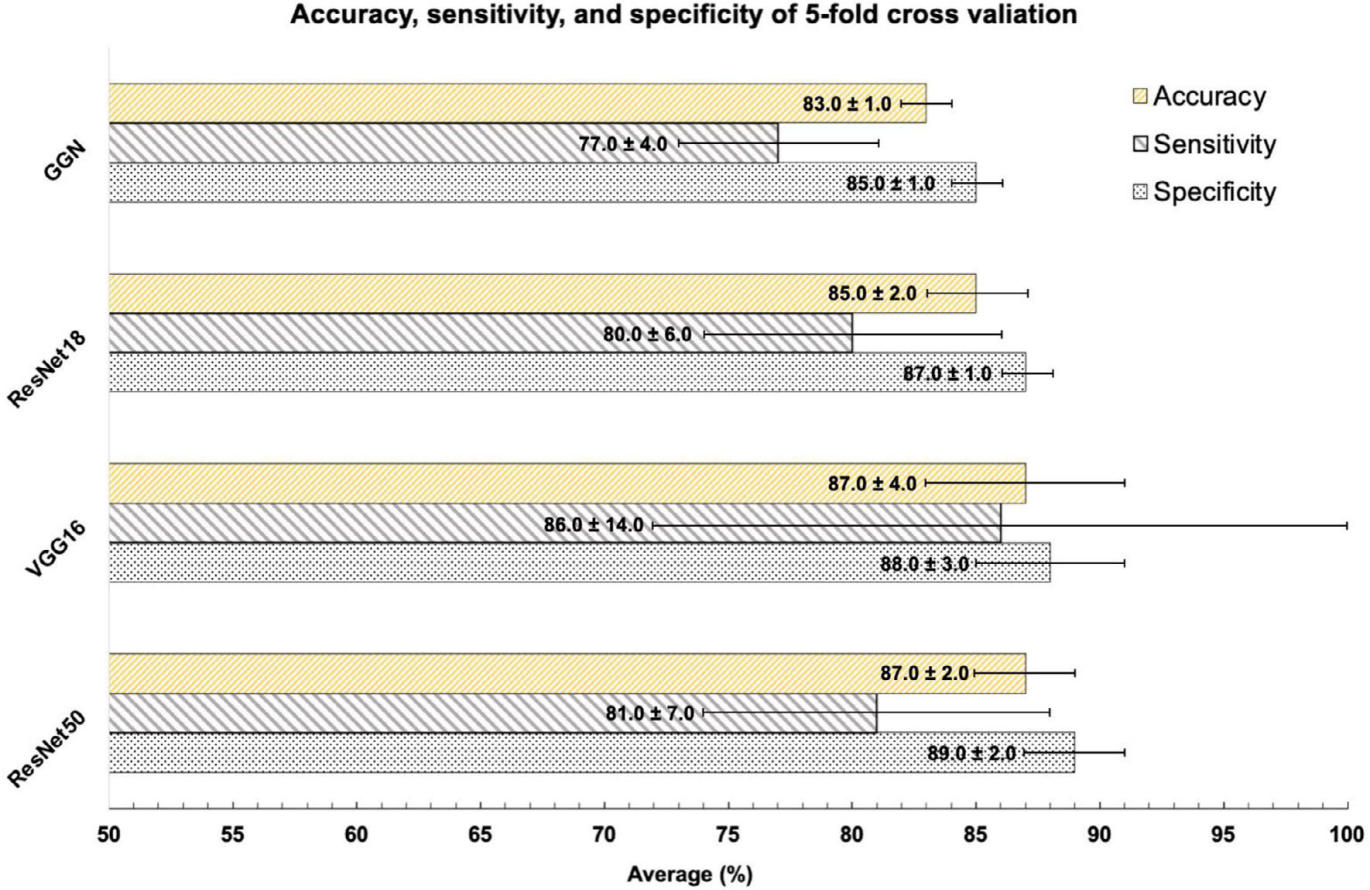
Presents a comparative analysis of model performance achieved through five‐fold cross‐validation in the training phase, utilizing the same dataset. The models under consideration are GGN, ResNet18, VGG16, and ResNet50.

Figure 5 presents a comparative analysis of four distinct deep learning architectures: ResNet50, GGN, ResNet18, and VGG16. The average model performance metrics, including accuracy and standard deviation (SD), are as follows: ResNet50 (87.0% ± 2.0%), GGN (83.0% ± 1.0%), ResNet18 (85.0% ± 2.0%), and VGG16 (87.0% ± 4.0%). In addition, sensitivity and specificity for these models are as follows: ResNet50 (81.0% ± 7.0% / 89.0% ± 2.0%), GGN (77.0% ± 4.0% / 85.0% ± 1.0%), ResNet18 (80.0% ± 6.0%), and VGG16 (85.0% ± 14.0% / 88.0% ± 3.0%). It is noteworthy that both ResNet50 and VGG16 exhibit the highest levels of accuracy. However, it is essential to highlight that VGG16 demonstrates significant variability in model performance when subjected to a five‐fold dataset cross‐validation, indicating a noteworthy degree of uncertainty.

Table 1 displays ResNet50's five‐fold cross‐validation performance during training. The ResNet50 model achieved an average accuracy of 0.87, with a standard deviation of 0.02, indicating consistent performance across folds. Sensitivity and specificity were at 0.81 and 0.89, respectively. The model's precision, recall, and F‐measure were 0.64, 0.81, and 0.71, respectively. The AUC value was consistently high at 0.98, suggesting strong discrimination between classes.

**TABLE 1:** The 5‐fold cross‐validation results of the accuracy, sensitivity, and specificity of ResNet50 in the training phase.

Fold	Accuracy	Sensitivity	Specificity	Precision	Recall	F‐measure	AUC
1	0.89	0.87	0.90	0.68	0.87	0.76	1.00
2	0.85	0.80	0.86	0.59	0.80	0.68	0.96
3	0.87	0.80	0.89	0.65	0.80	0.72	0.97
4	0.89	0.87	0.89	0.67	0.87	0.75	1.00
5	0.85	0.70	0.89	0.62	0.70	0.66	0.99
Average	0.87	0.81	0.89	0.64	0.81	0.71	0.98
SD	0.02	0.07	0.02	0.04	0.07	0.04	0.02

Abbreviation: AUC, area under ROC curve.

### 3.3 Comparison with previously published studies

Table 2 presents a comparative analysis between our ResNet50 model and previous research in the field of computer‐aided liver lesion diagnosis. Our study demonstrates the superiority of the ResNet50 model over a binary class Feedforward Neural Network (FFNN) model proposed by Hwang et al.^20^ in the classification of HMG and HCC. Our ResNet50 model achieved a sensitivity of 81.3% ± 3.8% for HMG and 80.7% ± 6.8% for HCC, as opposed to their study's 40% sensitivity. The model also achieved a specificity of 86.3% ± 7.6% for HMG and 81.2% ± 3.7% for HCC, surpassing their study's specificity of 60%. Furthermore, our model demonstrated an accuracy of 87.2% ± 2.2%, outperforming their study's accuracy of 51%.

**TABLE 2:** The comparison of predictive ResNet50 model's performance across previous studies.

Authors	Models	No. of original images	Number of classes	Sensitivity (%)	Specificity (%)	Accuracy (%)
Present study	CNN‐ResNet50 (Testing phase)	non‐FLL = 150	5	92.7 ± 7.2(non‐FLL)	89.3 ± 4.6(non‐FLL)	87.2 ± 2.2
		Cyst = 150		92.7 ± 2.8 (Cyst)	98.6 ± 2(Cyst)	
		FFS = 77		88.7 ± 6.9 (FFS)	81.6 ± 5.8(FFS)	
		HMG = 150		81.3 ± 3.8 (HMG)	86.3 ± 7.6(HMG)	
		HCC = 54		80.7 ± 6.8 (HCC)	81.2 ± 3.7 (HCC)	
Hwang et al. (2015)^20^	Two‐layered feed‐forward neural network (FFNN)	Cyst = 29 HMG = 37 Malignancy (HCC+MLC) = 33	2(Cyst vs. HMG, Cyst vs.Malignancy, HMG vs. Malignancy)	98 (Cyst vs. HMG) 97 (Cyst vs. Malignancy) 40 (HMG vs. Malignancy)	98 (Cyst vs. HMG) 98 (Cyst vs. Malignancy) 60 (HMG vs. Malignancy)	98 (Cyst vs. HMG) 98 (Cyst vs. Malignancy) 51 (HMG vs. Malignancy)
Reddy et al. (2018)^21^	Model1: CNN Model2: VGG16+transfer learning Model 3: VGG16+transfer learning+fine tuning	Normal liver = 64 and Fatty liver = 93	2	89 (1^st^ model) 95 (2^nd^ model) 95 (3^rd^ model)	85 (1^st^ model) 76 (2^nd^ model) 85 (3^rd^ model)	84 (1^st^ model) 88 (2^nd^ model) 91 (3^rd^ model)
Yamakawa et al. (2019)^22^	CNN‐based VGGnet	Cyst = 159 HMG = 68 HCC = 73 MLC = 24	4	98 (Cyst) 87 (HMG) 86(HCC) 46 (MLC)	12	88
			2 (Benign vs. Malignancy)	94 (Malignancy)	5	91
Ryu et al. (2021)^23^	CNN+ReLU	Cyst = 1214 HMG = 1220 HCC = 874 MLC = 1001	4	94 (Cyst) 83 (HMG) 67 (HCC) 82 (MLC)	90	80
			2 (Benign vs. Malignancy)	87 (Malignancy)	89	90
Nishida et al. (2022)^24^	CNN‐based VGGNet (Model 3, utilizing the largest available HCC dataset)	HCC = 1750 MLC = 396 HMG = 433 Cyst = 43	4	67.5 (HCC)	96 (HCC)	93.4 (HCC)

Abbreviations: CCN, convolution neural network; Cyst, simple hepatic cyst; FFS, fat focal sparing; HCC, hepatocellular carcinoma; HMG, hemangioma; MLC, metastases liver cancer; non‐FLL, non‐focal liver lesion.

In the classification of non‐Focal Liver Lesions (FLL) or fatty liver, our ResNet50 model demonstrated superior performance compared to conventional Convolutional CNNs and was comparable to the VGG16 model as reported by Reddy et al.^21^

Comparing the performance of our ResNet50 model to the CNN‐based VGGnet model developed by Yamakawa et al.^22^ in classifying HMG and HCC, both models achieved sensitivities over 80%. However, the sensitivity of ResNet50 notably surpassed that of the CNN‐based VGGnet (86.3 ± 7.6 (HMG) and 81.2 ± 3.7 (HCC) compared to their sensitivity of 12%).

When comparing our ResNet50 model to the CNN + ReLU model introduced by Ryu et al.,^23^ our model demonstrated superior performance in discerning HCC. Specifically, our model achieved an accuracy of 87.2% ± 2.2, sensitivity of 80.7% ± 6.8, and specificity of 81.2% ± 3.7, surpassing the results reported in their study, which showed accuracy, sensitivity, and specificity of 80%, 67%, and 90%, respectively.

While the VGGNet model proposed by Nishida et al.^24^ exhibited superior accuracy and specificity in HCC classification compared to ResNet50, ResNet50 demonstrated significantly greater sensitivity (80.7% ± 6.8% vs. 67%).

These results indicate the promising performance of the ResNet50 model in the context of liver lesion diagnosis, particularly in comparison to other established models.

## 4 DISCUSSION

In this study, we employed a pretrained ResNet50 deep learning model that was specifically optimized to classify five distinct classes of focal liver lesions (FLLs) from ultrasound images. This transfer learning approach reduces data requirements and computational time compared with training from scratch, while producing a network optimized for our specific task. Our model performed well on the training phase with an average accuracy of 87.0 %.

To assess the performance of the ResNet50, GGN, ResNet18, and VGG16 models, we conducted a rigorous five‐fold cross‐validation analysis on the same dataset, benchmarking it against other models. Our results demonstrated that our model consistently outperformed GGN and ResNet18. While VGG16 exhibited comparable performance to our model, it exhibited higher uncertainty (larger SD) in model accuracy and sensitivity across the five cross‐validation folds. Subsequently, we leveraged all models for predictive tasks in the subsequent phase across five folds, employing a pre‐divided testing dataset comprising 30 images per class in each fold. Consequently, our experiments established ResNet50 as the superior model among the options considered for comparison with other investigators.

We assessed the effectiveness of our newly optimized ResNet50 model by conducting performance evaluations on a testing subset, which comprised 10% of the data reserved for each fold. This subset consisted of 30 ultrasound images, encompassing both Focal Liver Lesions (FLLs) and non‐FLL images. The model achieved ranging from 90% to 96.7% accuracy for predicting cysts, correctly identifying 27−29 out of 30 images across five‐fold. In the remaining cases, the model misdiagnosed one image as non‐FLL and another one image as hemangiomas. These incorrect diagnoses likely reflect the shared hypoechogenic pattern among cysts and hemangiomas. Alternatively, they may be caused by posterior acoustic enhancement of the cystic lesion. Further investigation will be necessary to determine the specific reasons behind these misclassifications.

With regard to FFS, our model accurately predicted 23−28 out of 30 images with seven images being misclassified as non‐FLL, six images being misclassified as HMG, and five images being misclassified as HCC across five‐fold. This incorrect classification may be attributed to overlapping features of hypoechoic lesions that are present in both FFS and HCC. The factors contributing to this misclassification warrant further examination.

In the case of HCC, our model achieved ranging from 70%−86.7% accuracy, correctly identifying 21−26 out of 30 images. The remaining four HCC images were misclassified as FFS (8 images), HMG (12 images) and non‐FLL (9 images) across five‐fold, likely because hyperechoic HCCs share features with typical hemangiomas,^32^ FFS and liver tissue heterogeneity in non‐FFL. Inclusion of all HCC features (hypo, hyper, and mixed echogenicity) with large sample size during model training would enhance the performance of future deep learning models.

For hemangiomas, the model achieved ranging from 76.7% to 86.7% accuracy, correctly classifying 23−26 images out of 30 images. However, one image was misclassified as cysts, nine images as FFS, and 18 images as HCC across five‐fold. Hemangioma poses the greatest challenge for our model in misclassifying HMG to HCC, likely because its features overlap with other lesions. For example, typical hemangioma presents as a uniform hyperechogenic lesion with well‐defined margins, as may also be seen with small HCC. Additionally, atypical hemangiomas present various characteristics, such as inhomogeneous tissue and ill‐defined margins, that can be confused with HCC and hypoechoic lesions similar to FFS.^33^ Certain hemangiomas can exhibit posterior acoustic enhancement similar to hepatic cysts, potentially leading to misclassification by our model. Moreover, the inclusion of neighboring anatomical structures within the ROI, such as vessels or the gallbladder, may contribute to potential misclassification by the model. Additional research is necessary to understand and resolve this diagnostic ambiguity. Inclusion of the color dropper as a novel image feature may offer significant advantages in future investigations.

Our model achieved up to 100% (ranging from 83.3% to 100%) accuracy in non‐FLL classification with only one image being misclassified as Cyst, eight images as FFS, and three images as HCC for unclear reasons across five‐fold. It is possible that, because all ultrasound images in this study were obtained from chronic liver disease patients who met the inclusion criteria for the liver cancer screening project at the research site, the misinterpretation of non‐homogenous liver tissue in three patients led to this instance of misclassification. In addition, other tissue structures, such as hepatic vessels, in ROI might cause misclassification as cyst and FFS. The present study demonstrates promising results in ultrasound image classification; however, further investigation will be necessary to address the observed misclassifications, particularly when distinguishing hemangiomas from other lesions.

Previous studies^20^, ^21^, ^22^, ^23^, ^24^ indicate that deep learning models exhibit high efficacy in binary classification tasks, specifically when distinguishing between benign and malignant cases. However, results of this kind do not provide adequate support for routine clinical implementation. The extension to multiple classes addresses the practical challenges encountered in clinical scenarios, particularly in the context of ultrasound image‐based diagnosis of FLLs. In our study, we introduced a five‐class deep learning model for FLL diagnosis. Our model demonstrates superior performance in distinguishing HCC when compared with some existing approaches that utilize a four‐class methodology.

Our model achieved sensitivity and specificity values comparable to those reported by Hwang et al.,^20^ who utilized a binary class FFNN model that produced sensitivity/specificity values of 40%/60% for classifying HMG and HCC. Despite using more classes, our CNN‐ResNet50 model surpasses their reported values in terms of sensitivity, specificity, and accuracy. Our findings also align with those reported by Ryu et al.,^23^ who employed a CNN + ReLU model for classifying four classes of FLL (Cyst, HMG, HCC, and MLC) and two classes of FLLs (benign and malignant). The study by Nishida et al.,^24^ which relied on a large HCC dataset, also supports our findings. Despite having access to fewer HCC cases (54 images) compared with the models adopted by Ryu et al. and Nishida et al., our model achieved a sensitivity of 73.3%, surpassing their results of 67% and 67.5%, respectively. However, our model exhibited lower specificity and accuracy. This indicates that, compared with their models, ResNet50 was superior in diagnosing true positive cases, but inferior in diagnosing true negative cases.

Our model achieves lower performance (sensitivity, specificity, and accuracy) than reported by Yamakawa et al.^23^ for a CNN‐based VGGnet with four classes of FLLs (Cyst, HMG, HCC, and MLC) and two classes of FLL (benign and malignant). However, their model was limited in its ability to distinguish between MLC and other FLLs, with a sensitivity of only 46% and a specificity of 12%. The sensitivity and accuracy achieved by their model may be improved by adopting a binary class approach; however, specificity remains relatively low (5%), indicating poor performance for correctly identifying true negative cases. Additionally, our model achieved the highest performance in classifying non‐FLL from other FLLs, consistent with a study by Reddy et al.^21^ These authors utilized three different models (CNN, VGG16+transfer learning, and VGG16+transfer learning and fine‐tuning) for classifying normal liver and fatty liver tissue. Their results demonstrated good performance across all models, particularly the model that combined VGG16 with transfer learning and fine‐tuning.

This study presents several limitations. First, while the transfer learning network used here does not require an extensive dataset for training, the adopted sample size for FLLs was relatively small compared with the diverse lesion characteristics that are typically present in ultrasound images. A larger sample size may bolster ResNet50's performance in diagnosing FLLs; however, increasing sample size may not be sufficient. To address feature overlap among FFS, HMG, and HCC, future studies should include a broader spectrum of features for model training, including various characteristics of HCC and HMG such as hypo, hyper, and mixed echogenicity. Expert radiological assessment and assemblage of datasets with diverse image feature characteristics will significantly enhance model performance. Second, the absence of a consensus method for ultrasound image standardization led us to exclude image preprocessing for normalization. Future investigations should incorporate various image normalization techniques and compare their impact on model performance to determine the best method for image normalization. Third, the absence of MLC cases from our dataset may limit the clinical utility of our model. Future studies should include MLC cases to improve the applicability of ResNet50 to clinical settings. Finally, this study relied on data from a single center. Our findings therefore warrant further investigation through a multicenter study. Notwithstanding the above limitations, our five‐class FLLs‐trained model is adequate for developing CADx to assist in focal lesion screening, which is the primary purpose of the ultrasound modality.

## 5 CONCLUSION

The preliminary results show that the modified ResNet50 model adopted in this study produced satisfactory performance and accuracy values for most classes, particularly for non‐FLL, Cyst, and FFS cases, which were associated with higher sensitivity values. However, its ability to differentiate between HMG and HCC cases required the improvement, as evidenced by the high number of images misclassified as HCC. Compared with previous studies that employed four‐class deep learning approaches, ResNet50 outperformed CNN‐based ReLU and FFNN models in the diagnosis of HCC. Future investigations should thoroughly analyze the selected dataset with diverse FLL characteristics of HMG and HCC to gain a comprehensive understanding of model performance in diagnosis of these two FLLs.

## AUTHOR CONTRIBUTIONS

Guarantors of integrity of entire study, study concepts/study design, approval of final version of submitted manuscript, manuscript drafting or manuscript revision for important intellectual content, statistical analysis, Sutthirak Tangruangkiat and Monchai Phonlakrai; literature research, data acquisition or data analysis/interpretation, experimental studies, Napatsorn Chaiwongkot, Thanatcha Rawangwong, Araya Khunnarong, Chanyanuch Chainarong, Preyanun Sathapanawanthana, Pantajaree Hiranrat; and manuscript editing, agrees to ensure any questions related to the work are appropriately resolved, Chayanon Pamarapa, Ruedeerat Keerativittayayut, Witaya Sungkarat.

## CONFLICT OF INTEREST STATEMENT

The authors declare no conflict of interest.

## References

[1] Yang JD , Hainaut P , Gores GJ , et al. A global view of hepatocellular carcinoma: trends, risk, prevention and management. Nat Rev Gastroenterol Hepatol. 2019;16(10):589‐604. 31439937 10.1038/s41575-019-0186-y PMC6813818

[2] Arslanoglu A , Seyal AR , Sodagari F , et al. Current guidelines for the diagnosis and management of hepatocellular carcinoma: a comparative review. Am J Roentgenol. 2016;207(5):W88‐W98. 27490855 10.2214/AJR.15.15490

[3] Minami Y , Kudo M . Imaging modalities for assessment of treatment response to nonsurgical hepatocellular carcinoma therapy: contrast‐enhanced US, CT, and MRI. Liver Cancer. 2015;4(2):106‐114. 26697413 10.1159/000367733 PMC4682875

[4] Choudhary MM , Gupta A , Bena J , Emch T , Singh AD . Hepatic ultrasonography for surveillance in patients with uveal melanoma. JAMA Ophthalmol. 2016;134(2):174‐180. 26633182 10.1001/jamaophthalmol.2015.4810

[5] Dasarathy S , Dasarathy J , Khiyami A , Joseph R , Lopez R , McCullough AJ . Validity of real time ultrasound in the diagnosis of hepatic steatosis: a prospective study. J Hepatol. 2009;51(6):1061‐1067. 19846234 10.1016/j.jhep.2009.09.001 PMC6136148

[6] Ungtrakul T , Mahidol C , Chun‐On P , et al. Hepatocellular carcinoma screening and surveillance in 2293 chronic hepatitis B patients in an endemic area. World J Gastroenterol. 2016;22(34):7806‐7812. 27678364 10.3748/wjg.v22.i34.7806 PMC5016381

[7] Trepanier C , Huang A , Liu M , Ha R . Emerging uses of artificial intelligence in breast and axillary ultrasound. Clin Imaging. 2023;100:64‐68. 37243994 10.1016/j.clinimag.2023.05.007

[8] Shin J , Lee S , Yoon JK , Roh YH . Diagnostic performance of the 2018 EASL vs. LI‐RADS for hepatocellular carcinoma using CT and MRI: a systematic review and meta‐analysis of comparative studies. J Magn Reson Imaging. 2023;58(6):1942‐1950. doi:10.1002/jmri.28716 37010244

[9] Ruan S‐M , Huang H , Cheng M‐Q , et al. Shear‐wave elastography combined with contrast‐enhanced ultrasound algorithm for noninvasive characterization of focal liver lesions. Radiol Med (Torino). 2023;128(1):6‐15. 36525179 10.1007/s11547-022-01575-5

[10] Zhou LQ , Wang JY , Yu SY , et al. Artificial intelligence in medical imaging of the liver. World J Gastroenterol. 2019;25(6):672‐682. 30783371 10.3748/wjg.v25.i6.672 PMC6378542

[11] Sharma P , Suehling M , Flohr T , Comaniciu D . Artificial intelligence in diagnostic imaging: status quo, challenges, and future opportunities. J Thorac Imaging. 2020;35:S11‐S16. 32205816 10.1097/RTI.0000000000000499

[12] Milletari F , Navab N , Ahmadi S‐A , eds. V‐net: fully convolutional neural networks for volumetric medical image segmentation. 2016 fourth international conference on 3D vision (3DV), Stanford, CA, USA; 2016: 565‐571. doi:10.1109/3DV.2016.79

[13] Wang CJ , Hamm CA , Savic LJ , et al. Deep learning for liver tumor diagnosis part II: convolutional neural network interpretation using radiologic imaging features. Eur Radiol. 2019;29:3348‐3357. 31093705 10.1007/s00330-019-06214-8 PMC7243989

[14] Hamm CA , Wang CJ , Savic LJ , et al. Deep learning for liver tumor diagnosis part I: development of a convolutional neural network classifier for multi‐phasic MRI. Eur Radiol. 2019;29:3338‐3347. 31016442 10.1007/s00330-019-06205-9 PMC7251621

[15] Abbood AA , Shallal QM , Fadhel MA . Automated brain tumor classification using various deep learning models: a comparative study. Indones J Elect Eng Comput Sci. 2021;22(1):252.

[16] Jerbi F , Aboudi N , Khlifa N . Automatic classification of ultrasound thyroids images using vision transformers and generative adversarial networks. Scientific African. 2023;20:e01679.

[17] Tiyarattanachai T , Apiparakoon T , Marukatat S , et al. Development and validation of artificial intelligence to detect and diagnose liver lesions from ultrasound images. PLoS ONE. 2021;16(6):e0252882. 34101764 10.1371/journal.pone.0252882 PMC8186767

[18] Schmauch B , Herent P , Jehanno P , et al. Diagnosis of focal liver lesions from ultrasound using deep learning. Diagn Interv Imaging. 2019;100(4):227‐233. doi:10.1016/j.diii.2019.02.009 30926443

[19] Mostafiz R , Rahman MM , Islam AKMK , Belkasim S . Focal liver lesion detection in ultrasound image using deep feature fusions and super resolution. Mach Learn Knowl Extr. 2020;2(3):172‐191. doi:10.3390/make2030010

[20] Hwang YN , Lee JH , Kim GY , Jiang YY , Kim SM . Classification of focal liver lesions on ultrasound images by extracting hybrid textural features and using an artificial neural network. Biomed Mater Eng. 2015;26(s1):S1599‐S1611. 26405925 10.3233/BME-151459

[21] Reddy DS , Bharath R , Rajalakshmi P , eds. A novel computer‐aided diagnosis framework using deep learning for classification of fatty liver disease in ultrasound imaging. 2018 IEEE 20th international conference on e‐health networking, applications and services (Healthcom), Ostrava, Czech Republic; 2018:1‐5. doi:10.1109/HealthCom.2018.8531118

[22] Yamakawa M , Shiina T , Nishida N , Kudo M , eds. Computer aided diagnosis system developed for ultrasound diagnosis of liver lesions using deep learning. 2019 IEEE International Ultrasonics Symposium (IUS), Glasgow, UK; 2019:2330‐2333. Doi:10.1109/ULTSYM.2019.8925698

[23] Ryu H , Shin SY , Lee JY , Lee KM , H‐j Kang , Yi J . Joint segmentation and classification of hepatic lesions in ultrasound images using deep learning. Eur Radiol. 2021;31:8733‐8742. 33881566 10.1007/s00330-021-07850-9 PMC8523410

[24] Nishida N , Yamakawa M , Shiina T , et al. Artificial intelligence (AI) models for the ultrasonographic diagnosis of liver tumors and comparison of diagnostic accuracies between AI and human experts. J Gastroenterol. 2022;57(4):309‐321. 35220490 10.1007/s00535-022-01849-9 PMC8938378

[25] Zhou J , Sun H , Wang Z , et al. Guidelines for the diagnosis and treatment of hepatocellular carcinoma (2019 edition). Liver Cancer. 2020;9(6):682‐720. 33442540 10.1159/000509424 PMC7768108

[26] He K , Zhang X , Ren S , Sun J , eds. Deep Residual Learning for Image Recognition. 2016 IEEE Conference on Computer Vision and Pattern Recognition (CVPR); 2016:770‐778.

[27] Reddy ASB , Juliet DS , eds. Transfer learning with ResNet‐50 for malaria cell‐image classification. 2019 International Conference on Communication and Signal Processing (ICCSP) IEEE; 2019:0945‐0949.

[28] Al‐Haija QA , Adebanjo A , eds. Breast cancer diagnosis in histopathological images using ResNet‐50 convolutional neural network. 2020 IEEE International IOT, Electronics and Mechatronics Conference (IEMTRONICS) IEEE; 2020:1‐7.

[29] Rajpal S , Lakhyani N , Singh AK , Kohli R , Kumar NJC . Using handpicked features in conjunction with ResNet‐50 for improved detection of COVID‐19 from chest X‐ray images. Chaos, Solitons Fractals. 2021;145:110749. 33589854 10.1016/j.chaos.2021.110749 PMC7874964

[30] Walvekar S , Shinde DS , eds. Detection of COVID‐19 from CT images using resnet50. 2^nd^ International Conference on Communication & Information Processing (ICCIP); 2020.

[31] Alam S , Tomar NK , Thakur A , Jha D , Rauniyar A . Automatic polyp segmentation using u‐net‐resnet50; 2020.

[32] Mazzola G , Virdone R , Orlando A , et al. Evolution of the echographic picture in primary liver tumor associated with cirrhosis. Radiol Med. 1989;77(5):488‐492. 2546193

[33] Lin S , Zhang L , Li M , Cheng Q , Zhang L , Zheng S . Atypical hemangioma mimicking mixed hepatocellular cholangiocarcinoma: case report. Medicine (Baltimore). 2017;96(50):e9192. 29390333 10.1097/MD.0000000000009192 PMC5815745

